# Clinical and prognostic factors in pleural mesothelioma: insights from a Latin American cohort

**DOI:** 10.1007/s12094-026-04337-1

**Published:** 2026-04-30

**Authors:** Ricardo Brugés, Carlos Carvajal, Marcela Núñez, Alejandra Navarrete, Rafael Parra-Medina

**Affiliations:** 1 Departamento de Oncología Clínica, Instituto Nacional de Cancerología, Bogotá, Colombia; 2 Departamento de Cirugía de Tórax, Instituto Nacional de Cancerología y Fundación CTIC, Bogotá, Colombia; 3 Grupo de Investigación Clínica y Epidemiológica del Cáncer,, Instituto Nacional de Cancerología, Bogotá, Colombia; 4 Departamento de Patología Oncologica, Instituto Nacional de Cancerología, Bogotá, Colombia; 5 Instituto de Investigación, Universidad FUCS, Bogotá, Colombia

**Keywords:** Mesothelioma, Asbestos, Latin America, Colombia, Chemotherapy, Survival, Low- and middle-income countries

## Abstract

**Background:**

Pleural mesothelioma (PM) remains a lethal but preventable disease whose burden is shifting toward low- and middle-income countries (LMICs) where asbestos use has persisted or has only recently been banned. This study describes the clinical characteristics, management patterns, and prognostic factors of PM in a contemporary Colombian cohort.

**Methods:**

We conducted a retrospective cohort study of adults with histologically confirmed pleural mesothelioma diagnosed at a tertiary referral cancer center in Bogotá, Colombia, from January 1, 2011, to December 31, 2023. Overall survival (OS) was defined from the histologic diagnosis date to death from any cause; patients without a recorded death were censored at the last documented oncologic contact. Real-world progression-free survival (PFS) was defined from diagnosis to documented progression (radiologic or clinician-documented) or death, whichever occurred first; patients without progression or death were censored at last contact. Survival was estimated using Kaplan–Meier methods and modeled using Cox proportional hazards regression.

**Results:**

The cohort included 106 patients (mean age, 63.2 years; 73.6% male; 84.9% epithelioid histology). There were 105 deaths (1 OS censor). Median OS was 9.9 months (95% CI, 7.1–14.1), with 12- and 24 month OS of 46.2% and 12.3%. Real-world PFS included 105 events (1 PFS censor). Median PFS was 8.1 months (95% CI, 6.2–9.4), with 12- and 24 month PFS of 30.2% and 6.6%. In multivariable analyses focused on baseline prognostic factors, ECOG performance status 2–3 was associated with shorter OS and PFS.

**Conclusions:**

In this contemporary Colombian cohort, OS and PFS were short, and baseline functional status was strongly associated with outcomes. Treatment-stratified survival patterns are reported descriptively and should not be interpreted as comparative effectiveness. These data add regional evidence for pleural mesothelioma prognosis in routine practice.

**Supplementary Information:**

The online version contains supplementary material available at https://doi.org/10.1007/s12094-026-04337-1.

## Introduction

Pleural mesothelioma (PM) is an aggressive tumor strongly associated with asbestos exposure and remains a global clinical and public-health challenge [1, 2]. Despite advances in multimodal care—including surgery, platinum–pemetrexed chemotherapy, and immune checkpoint inhibitors—median survival remains limited in most real-world settings [3]. Contemporary practice guidelines recommend nivolumab plus ipilimumab or platinum–pemetrexed-based regimens as first-line options for unresectable disease [4]. In Latin America, patterns of industrial asbestos use, regulation, and exposure differ widely: only a few countries have enacted complete bans, and use has persisted or only recently declined [5]. In Colombia, Law 1968 of 2019 (“Ley Ana Cecilia Niño”) banned asbestos production and use in January 2021, yet the long latency of PM implies that disease burden will continue for decades.

Prognosis in PM depends on clinical and pathological variables such as age, performance status, and histology [2]. Pleural mesothelioma is histologically classified into three major subtypes: epithelioid (EM), biphasic (BM), and sarcomatoid (SM). Despite advances in diagnosis and treatment, overall prognosis remains poor, with median survival typically ranging from 9 to 12 months and a 5 year survival rate of roughly 5%. Among these variants, EM generally confers a more favorable outcome than BM and SM. Emerging evidence also indicates that prognosis within PM is shaped not only by broad histologic subtype but also by more nuanced features, including specific architectural patterns and the histologic grading of epithelioid tumors.

In recent years, immunotherapy has become part of the standard first-line management for unresectable pleural mesothelioma. In the 3 year update of CheckMate 743, nivolumab plus ipilimumab improved overall survival compared with platinum–pemetrexed chemotherapy [6]. Several combination strategies and novel agents remain under clinical evaluation. Together, these developments reflect gradual but meaningful progress toward more effective and individualized treatment options for patients with malignant pleural mesothelioma (MPM). However, most prognostic evidence originates from high-income countries and published Latin American cohorts remain limited [7]. Moreover, regional heterogeneity in asbestos exposure and health-system capacity may modify both presentation and survival, but these aspects are underexplored. Consequently, this study aimed to describe prognostic patterns of PM in Colombia, analyzing clinical, pathological, inflammatory, and immunologic factors in relation to survival across treatment modalities (chemotherapy, immunotherapy, surgery, and radiotherapy).

## Methods

### Study design and setting

We conducted a retrospective, single-center cohort study at the Instituto Nacional de Cancerología (INC), Bogotá, Colombia, a national tertiary oncology referral hospital. INC operates within Colombia’s national health system, which includes contributory, subsidized, and special coverage regimes. The study protocol (version 02, November 30, 2023) was approved by the INC Research and Ethics Committee and classified as minimal risk because it used existing clinical data and archived tissue specimens.

### Study population

Eligible participants were adults (≥ 18 years) with histologically confirmed pleural mesothelioma seen in thoracic surgery or medical oncology between January 1, 2011, and December 31, 2023. For survival analyses, the analytic data cutoff was defined as the latest observed follow-up date in the dataset (the maximum of the last institutional oncologic contact and recorded death dates), which was August 10, 2024.

We recorded data from formalin-fixed, paraffin-embedded tumor blocks or unstained slides from primary or metastatic tissue when available. Blocks were reviewed by an experienced thoracic pathologist to confirm tumor content and suitability for additional staining. Specimens were not studied further when insufficient residual tissue could jeopardize future diagnostic requests, but patients were included in the analysis if at least one biomarker result was available.

### Measurements and exposures

Baseline characteristics such as age, sex, asbestos exposure history, comorbidities, Eastern Cooperative Oncology Group (ECOG) performance status, tumor histology and grade, AJCC 8th edition stage, and treatment modalities (surgery, chemotherapy, radiotherapy, immune checkpoint inhibitors) were recorded. Laboratory covariates (hemoglobin, albumin, lactate dehydrogenase, and leukocyte differentials) closest to diagnosis were recorded. A reclassification of tumor grade was conducted to adhere to updated WHO classification recommendations [8].

### Outcomes

The primary outcome was overall survival (OS), defined as the interval from the diagnostic pathology date to death from any cause. Patients without a recorded death were censored at the date of last documented oncologic contact.

The secondary outcome was real-world progression-free survival (PFS), defined from diagnosis to the first documentation of disease progression (radiologic report or clinician-documented progression in oncology notes) or death, whichever occurred first. Patients without progression or death were censored at the last oncologic contact. One patient met the criteria for administrative censoring for PFS (no documented progression and alive at last contact).

### Data handling and statistical analysis

All data were extracted from the electronic medical record into a secure REDCap database by trained staff with double-entry verification and quality checks at analysis. Analyses were performed in R version 4.5.2 and Stata 17, using 2-sided *p* < 0.05. Categorical variables are summarized as counts and percentages; continuous variables are summarized as mean (SD) or median (IQR), as appropriate.

OS and PFS were estimated using the Kaplan–Meier method and compared using log-rank tests; Kaplan–Meier curves are presented with numbers at risk. Confidence intervals for Kaplan–Meier estimates were computed using a log-minus-log transformation. Associations between baseline prognostic factors (age, sex, ECOG category, clinical stage, histologic subtype, and platelet category) and OS/PFS were evaluated using Cox proportional hazards models. Age was evaluated for potential nonlinearity using flexible specifications; because results were consistent with a linear effect in the baseline models, age is presented as a 1 year increase in Table 3. Treatment-inclusive models were considered exploratory because of confounding by indication and were used only for sensitivity analyses. Proportional hazards assumptions were evaluated using Schoenfeld residuals. There was some evidence of nonproportional hazards for age and clinical stage in OS models and for age in PFS models; adjusted hazard ratios are therefore interpreted as average effects over follow-up (Supplementary Table S1).

Missingness was summarized for each variable of interest. Primary regression models used complete-case analysis for covariates included in each model; variables with substantial missingness (e.g., lactate dehydrogenase and C-reactive protein) were not included in primary prognostic models. No imputation was performed.

### Ethics

The study complied with the Declaration of Helsinki, CIOMS guidelines and Colombian Resolution 8430/1993. Because it used retrospective de-identified data, informed consent was waived by the INC Ethics Committee.

## Results

### Baseline characteristics

A total of 106 patients with histologically confirmed pleural mesothelioma were included (see Table 1). The mean age was 63.2 ± 10.2 years, and 73.6% (*n* = 78) were male. Approximately half of the cohort was ≥ 65 years (48.1%) and most were covered by either the subsidized (50.0%) or contributory (48.1%) health-insurance schemes. Nearly 70% of patients resided in Bogotá D.C., reflecting the referral pattern of the study site.

**Table 1:** Baseline clinical characteristics

Variable	All patients (*n* = 106)
Demographics	
Age (years)	
Mean ± SD	63.2 ± 10.2
Age groups, *n* (%)	
< 65	55 (51.9)
≥ 65	51 (48.1)
Sex, *n* (%)	
Female	28 (26.4)
Male	78 (73.6)
Health insurance scheme, *n* (%)	
Subsidized	53 (50.0)
Contributory	51 (48.1)
Special	1 (0.9)
Uninsured	1 (0.9)
Place of residence, *n* (%)	
Bogotá D.C	74 (69.8)
Sibaté	7 (6.6)
Other	25 (23.6)
Medical history	
Comorbidities, *n* (%)	
Yes	55 (51.9)
No	51 (48.1)
Exposure history, *n* (%)	
Asbestos	16 (15.1)
Tobacco	28 (26.4)
Asbestos and tobacco	29 (27.4)
None	33 (31.1)
Family history, *n* (%)	
Yes	4 (3.8)
No	102 (96.2)
Clinical characteristics	
Body Mass Index (BMI)	
Mean ± SD	23.2 ± 3.91
ECOG performance status, *n* (%)	
0	16 (15.1)
1	63 (59.4)
2	22 (20.8)
3	5 (4.7)
Tumor laterality, *n* (%)	
Right	55 (51.9)
Left	51 (48.1)
TNM classification, *n* (%)	
IA	3 (2.8)
IB	4 (3.8)
II	7 (6.6)
IIIA	10 (9.4)
IIIB	23 (21.7)
IV	56 (52.8)
No information	3 (2.8)
Clinical stage, *n* (%)	
Early	7 (6.6)
Locally advanced	40 (37.7)
Metastatic	56 (52.8)
No information	3 (2.8)
Histologic type, *n* (%)	
Biphasic	8 (7.5)
Epithelioid	90 (84.9)
Sarcomatoid	8 (7.5)
Histologic grade†, *n* (%)	
High	39 (43.3)
Low	26 (28.9)
Missing data	25 (27.7)
Hemoglobin (g/dL), median (IQR)	13.7 [11.7–24.0]
Platelets (10^3^/µL), median (IQR	400.0 [315.5–508.0]
LDH (U/L), median (IQR	217.0 [157.8–349.8]
C-reactive protein, median (IQR	13.1 [5.10–23.6]

This table describes the baseline characteristics of the included patients, including demographics, medical history and clinical characteristics

Regarding medical history, 52% of patients had at least one comorbidity, most commonly hypertension (35%) and type 2 diabetes mellitus (22%). Only one patient (0.9%) had chronic obstructive pulmonary disease. A history of asbestos exposure was documented in 15.1%, tobacco use in 26.4%, and combined asbestos + tobacco exposure in 27.4%, for a total of 68.9% of patients with a history of asbestos or tobacco exposure in the cohort. A family history of cancer was recorded in 4%.

Baseline clinical status showcased 75% of patients with an ECOG performance status of 0 or 1, and 52.8% with stage IV disease according to the TNM classification. Clinical stage was locally advanced in 38%, and metastatic in 53% of patients. Epithelioid histology was predominant (85%), followed by biphasic (7.5%) and sarcomatoid (7.5%) types. At least 43% of patients had high histologic grade, as grade was not available for 25 patients (28%).

### Treatments received

The median (IQR) time from diagnosis to treatment initiation was 3.53 (2.0–4.6) months. For those undergoing surgery, the interval was 2.44 (2.0–5.0) months, and for patients receiving palliative chemotherapy it was 3.48 (1.6–3.9) months. Eighty-five patients (80.2%) received active oncologic therapy with palliative chemotherapy in 59 (55.6%), extrapleural pneumonectomy (EPP) in 10 (9.4%), and best supportive care in 21 (19.8%) (see Table 2).

**Table 2:** Treatments received

Treatment received	All patients (*n* = 106)
	*n*(%)
Surgery	11 (10.4)
Type of surgery	
Extrapleural pneumonectomy	10 (90.9)
Decortication and pleurectomy	1 (9.1)
Chemotherapy	
Yes	68 (64.1)
Not received	14 (13.2)
Not prescribed	24 (22.6)
Type of chemotherapy	
Neoadjuvant (*n* = 1)*	
Pemetrexed	1 (100)
Adjuvant (*n* = 7)*	
Cisplatin (CDDP) + Pemetrexed	7 (100)
Palliative (*n* = 60)*	
First-line treatment	
Cisplatin (CDDP) + Pemetrexed	31 (51.7)
Carboplatin + Pemetrexed	10 (16.6)
CDDP + Pemetrexed – Carboplatin + Pemetrexed	4 (6.66)
CDDP + Pemetrexed + Bevacizumab	1 (1.67)
First/Second-line treatment	
CDDP + Pemetrexed/Gemcitabine	5 (8.33)
Carboplatin + Pemetrexed/Gemcitabine	3 (5.00)
CDDP + Pemetrexed/Gemcitabine + Pemetrexed	2 (3.33)
CDDP + Pemetrexed − Carboplatin + Pemetrexed/Gemcitabine	1 (1.67)
First/Second/Third line treatment or more	
CDDP + Pemetrexed/Gemcitabine/Vinorelbine	1 (1.67)
CDDP + Pemetrexed/Gemcitabine + Carboplatin/Vinorelbine	1 (1.67)
Other	1 (1.67)
Radiotherapy	21 (19.8)
Palliative care	26 (54.7)

The table describes the treatments received by the included patients during their clinical follow-up

### Follow-up and survival outcomes

Because censoring was minimal for OS (1 censored patient), the median follow-up estimated by reverse Kaplan–Meier was not estimable. We report the last observed date (August 10, 2024), the proportion of events, and numbers at risk at 12 and 24 months alongside Kaplan–Meier estimates to document data maturity. Fifty-six patients (52.8%) had documented progression during follow-up. The 12- and 24 month OS rates were 46.2% (95% CI, 37.6–56.8) and 12.3% (95% CI, 7.4–20.4), respectively, with a median OS of 9.9 months (95% CI, 7.1–14.1). The 12- and 24 month PFS rates were 30.2% (95% CI, 22.6–40.3) and 6.6% (95% CI, 3.2–13.5), respectively, with a median PFS of 8.1 months (95% CI, 6.2–9.4).

### Survival by clinical and laboratory variables

**Fig. 1:**
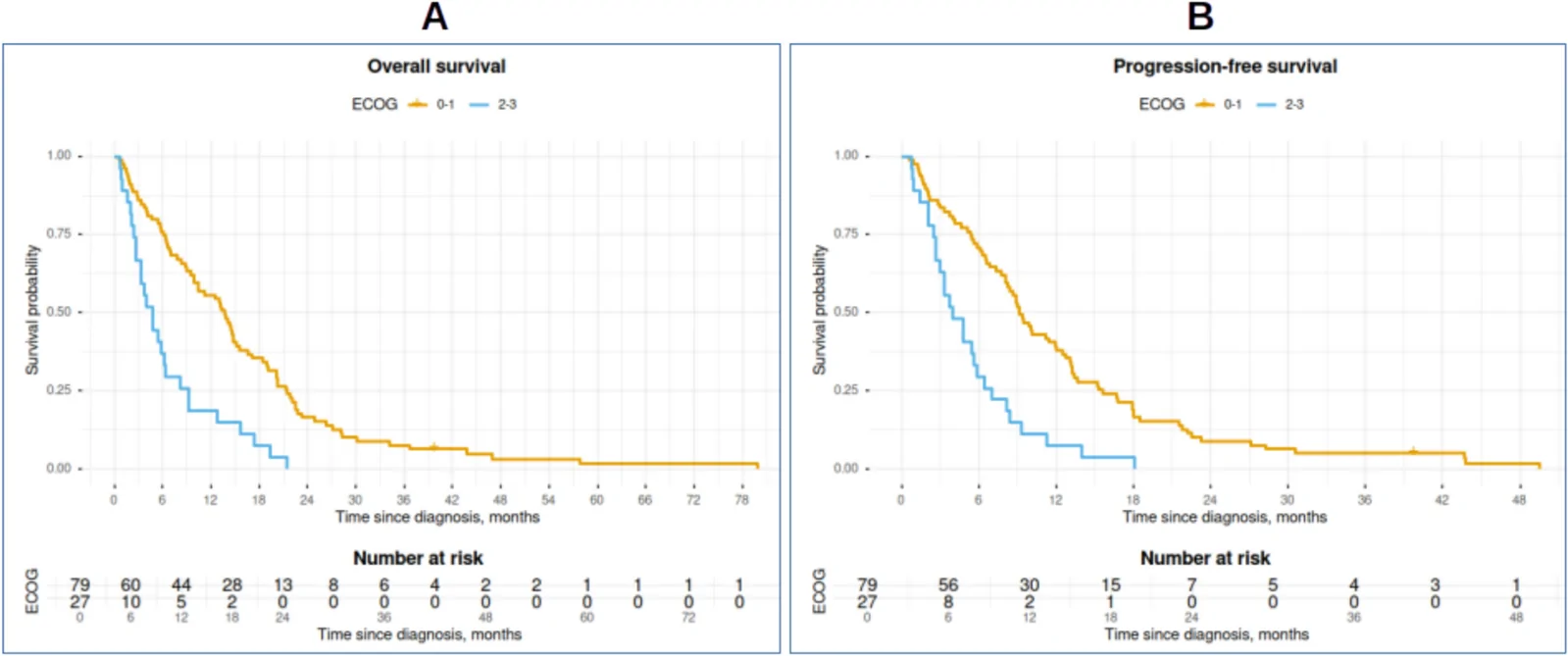
Kaplan–Meier estimates of overall survival (OS) and progression-free survival (PFS) in pleural mesothelioma stratified by ECOG performance status (0–1 vs 2–3). **A** OS and **B** PFS stratified by ECOG 0–1 versus ECOG 2–3. Curves are unadjusted and shown with numbers at risk. *ECOG* Eastern Cooperative Oncology Group

**Fig. 2:**
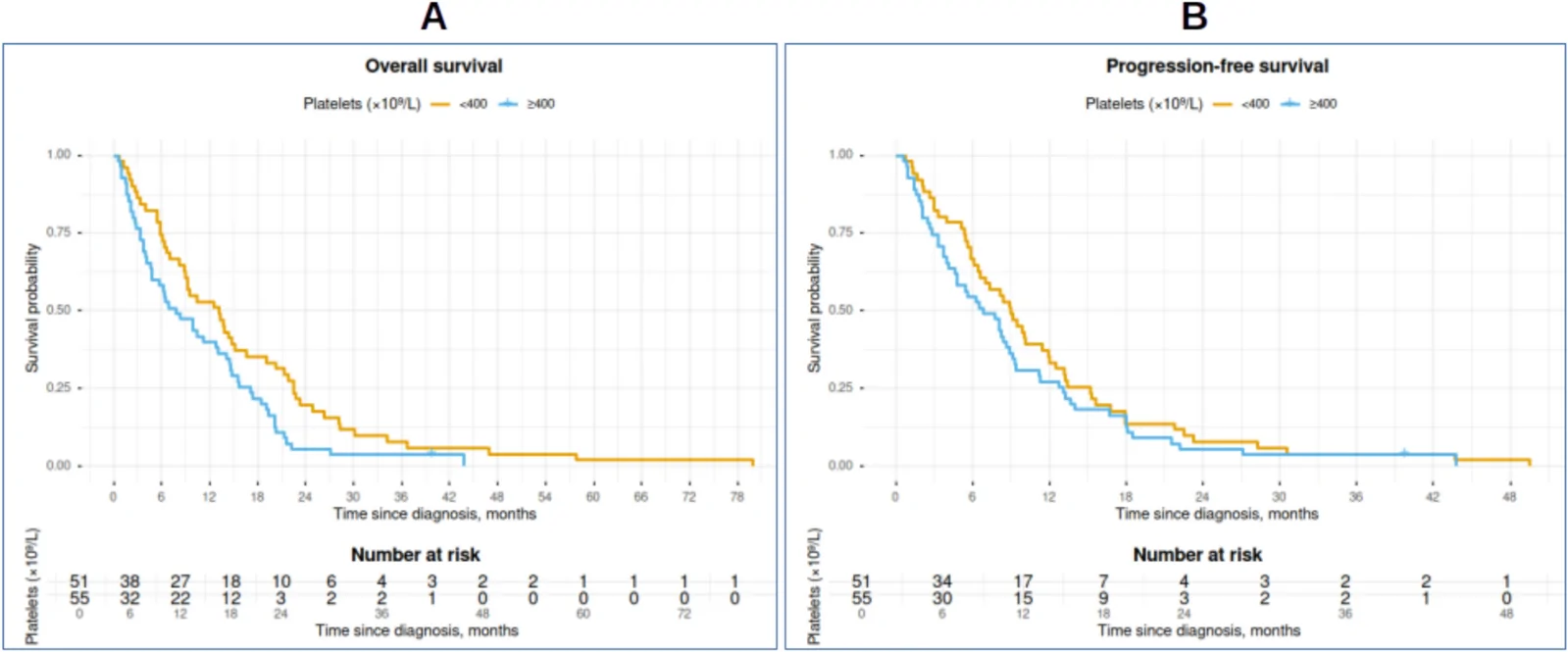
Overall survival (OS) and progression-free survival (PFS) by platelet count category. **A** OS and **B** PFS stratified by platelet count < 400 versus ≥ 400. Curves are unadjusted and shown with numbers at risk

In unadjusted Kaplan–Meier analyses, ECOG performance status and platelet count categories were associated with OS and PFS. Patients with ECOG 0–1 had longer OS than those with ECOG 2–3 (median OS, 13.7 vs 4.8 months; log-rank *P* < 0.001) (Fig. 1). ECOG 0–1 was also associated with longer PFS (Fig. 1). Given very small subgroup sizes for non-epithelioid histology and substantial missingness for selected inflammatory markers, subgroup analyses for histology and lactate dehydrogenase were treated as exploratory and are not presented as primary figures (Fig. 2).

### Survival by primary treatment (descriptive)

**Fig. 3:**
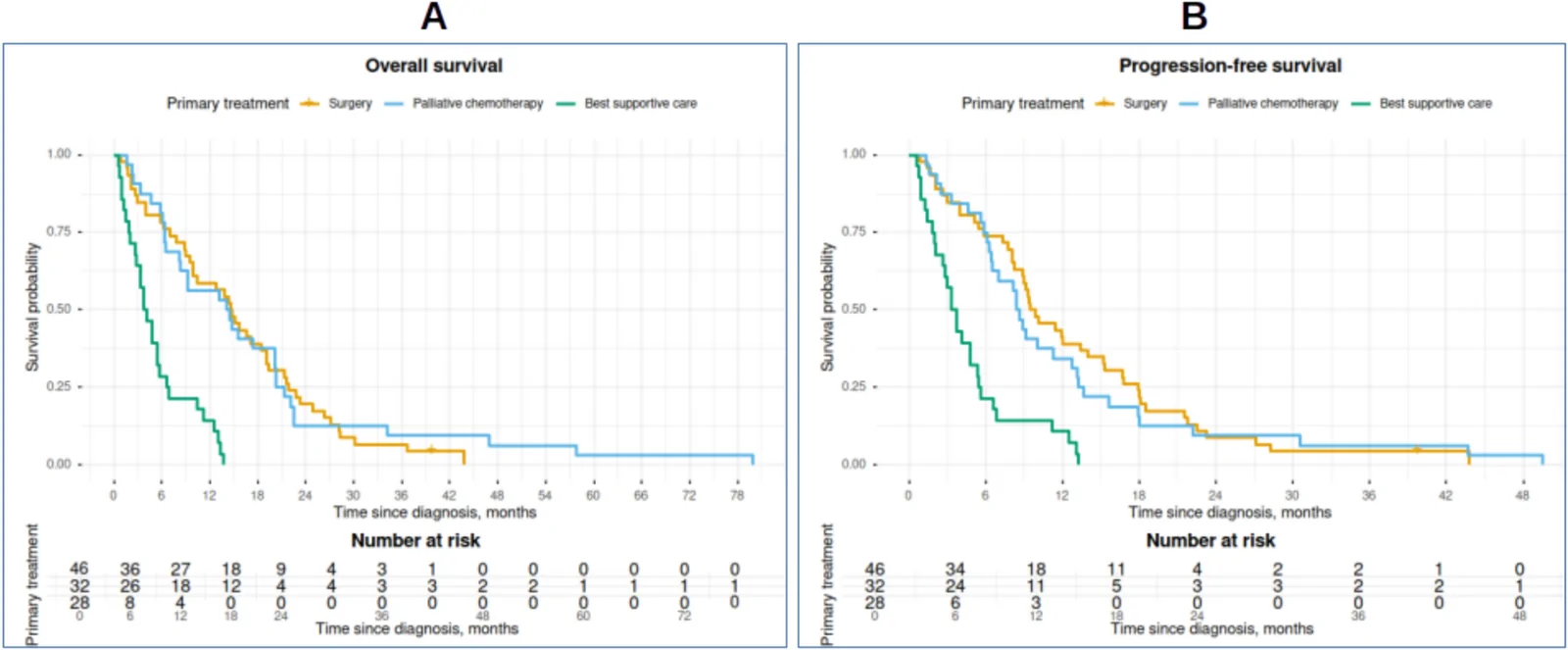
Descriptive Kaplan–Meier curves by primary treatment. **A** OS and **B** PFS are stratified by primary treatment (surgery, palliative chemotherapy, best supportive care). Curves are unadjusted and shown with numbers at risk. Because treatment groups differ in baseline performance status and disease extent, these curves are descriptive and should not be interpreted as comparative effectiveness

Kaplan–Meier curves stratified by primary treatment are presented descriptively (Fig. 3). Patients receiving best supportive care differed clinically from those receiving systemic therapy or surgery, so these curves should not be interpreted as comparative effectiveness. Because the surgical subgroup was small, we do not frame the absence of separation between surgery and palliative chemotherapy curves as evidence of equivalence.

### Multivariable analysis

**Fig. 4:**
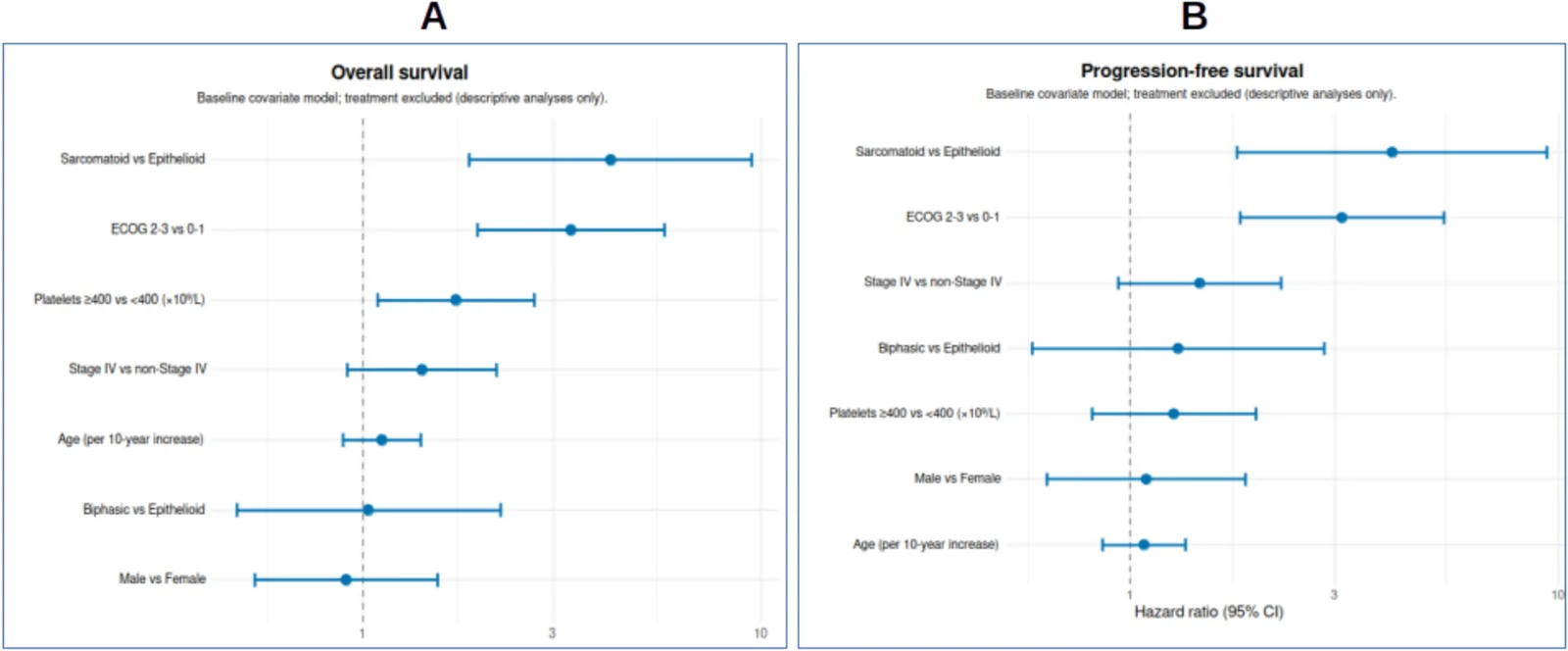
Multivariable Cox models for baseline prognostic factors. Forest plots show adjusted hazard ratios (HRs) and 95% confidence intervals for **A** overall survival (OS) and **B** progression-free survival (PFS). Models include baseline covariates selected a priori (age, sex, ECOG category, clinical stage, histologic subtype, and platelet category) and exclude treatment variables due to confounding by indication. Proportional hazards assumptions were evaluated using Schoenfeld residuals (global tests in Supplementary Table S1). *ECOG* Eastern Cooperative Oncology Group

In baseline prognostic models excluding treatment variables (Fig. 4; Table 3), ECOG performance status 2–3 was associated with shorter OS and PFS. Sarcomatoid histology was also associated with shorter OS and PFS, while thrombocytosis (platelets ≥ 400 × 10^9/L) was associated with shorter OS but not PFS. Given small nonepithelioid subgroups, histology estimates are interpreted cautiously.

**Table 3:** Multivariable Cox proportional hazards models for baseline prognostic factors

Covariate	Adjusted HR (95% CI), OS	*P* value (OS)	Adjusted HR (95% CI), PFS	*P* value (PFS)
Age (per 1 year increase)	1.01 (0.99–1.03)	0.331	1.01 (0.99–1.03)	0.512
Male vs Female	0.91 (0.54–1.54)	0.723	1.09 (0.64–1.86)	0.750
ECOG 2–3 vs 0–1	3.33 (1.94–5.73)	<.001	3.12 (1.81–5.39)	<.001
Non–stage IV vs stage IV	0.71 (0.46–1.09)	0.120	0.69 (0.44–1.06)	0.093
Biphasic vs Epithelioid	1.03 (0.48–2.21)	0.934	1.29 (0.59–2.84)	0.521
Sarcomatoid vs Epithelioid	4.19 (1.85–9.49)	<.001	4.09 (1.78–9.41)	<.001
Platelets ≥ 400 × 10^9/L vs < 400 × 10^9/L	1.71 (1.09–2.69)	0.020	1.26 (0.81–1.96)	0.297

OS was defined as the time from histologic diagnosis to death from any cause; patients without a recorded death were censored at the last documented oncologic contact. PFS was defined as the time from diagnosis to documented progression (radiologic or clinician-documented) or death, whichever occurred first; patients without progression or death were censored at the last contact. Models include baseline covariates only and exclude treatment variables because of confounding by indication. Analyses were conducted using complete-case data for included covariates. ECOG indicates Eastern Cooperative Oncology Group

## Discussion

### Summary of findings

Pleural mesothelioma (PM) poses major diagnostic and therapeutic challenges despite significant advances in multimodal management. In routine care settings, functional status and access to therapy often correlate with broader social and health-system factors; our dataset does not directly measure care intervals or authorization delays, so we describe these considerations as contextual rather than causal.

Median OS in this cohort was within the range reported in several contemporary series, but direct comparisons across health systems should be interpreted cautiously because this study did not include an external comparator cohort. In our data, treatment-stratified Kaplan–Meier curves are presented descriptively; differences across treatment groups are expected to reflect baseline status and disease extent rather than comparative effectiveness. The surgical subgroup was small, so the lack of separation between curves should not be interpreted as equivalence.

Although INC is a tertiary referral cancer center, the British Thoracic Society guideline defines a specialist mesothelioma multidisciplinary team as one that manages at least 25 mesothelioma cases per year [9]. This caseload threshold is rarely met in Latin America, which limits procedural volume and specialized pathways.

### Prognostic disparities

While ECOG performance status and treatment access are well-recognized determinants of outcome in mesothelioma [10, 11], their effect appears more pronounced in middle-income contexts, where they reflect not only biological frailty but also health-system limitations. As such, deterioration in ECOG often results from diagnostic delays, malnutrition, and socioeconomic vulnerability rather than disease burden alone [12]. The pronounced survival disadvantage in patients with ECOG ≥ 2 or limited access to therapy in our results highlights the structural and socioeconomic barriers that define cancer care in middle-income settings, particularly notable in our cohort where half of the patients were part of a subsidized healthcare coverage regimen indicative of a population with greater socioeconomic disadvantages.

Similarly, access to systemic therapy acts as a structural variable: in Colombia and neighboring countries, treatment initiation is often delayed by insurance authorization processes, geographic fragmentation, or cost-sharing barriers, producing longer pre-treatment intervals—median 3.5 months in our cohort—than are typically reported in Europe or North America [11–13]. Because the diagnosis is often delayed, a high portion of patients remain with an “unspecified mesothelioma” diagnosis due to limited immunohistochemistry and staging tools, which further explains why many cases present at an advanced stage or do not receive therapy [14]. These factors reflect both industrial history and current health-system shortcomings.

### Therapeutic landscape and regional constraints

Recent literature reinforces that the global management of MPM is rapidly evolving, particularly in diagnostic precision and patient selection for multimodal therapy. Advances in immunohistochemistry and molecular pathology—including the use of BAP1 loss as a highly specific marker in appropriate diagnostic settings—have improved diagnostic accuracy and helped distinguish mesothelioma from reactive mesothelial proliferations and metastatic adenocarcinoma [8, 15]. The implementation of next-generation sequencing (NGS) panels, alongside BAP1 and NF2 mutation profiling, also provides insight into tumor biology and potential therapeutic vulnerabilities [16]. Moreover, the increasing use of PET/CT and MRI with diffusion sequences enhances staging accuracy, and artificial intelligence-based radiomic models are emerging as tools to predict histologic subtype and prognosis [17]. These diagnostic improvements are still limited in Latin America due to cost, infrastructure, and pathologist training gaps, but they represent feasible targets for regional capacity building.

The role of surgery in PM remains highly debated. Although historical series suggested a potential benefit of P/D within multimodal therapy, contemporary randomized evidence has challenged these assumptions. The descriptive similarity between surgery-containing pathways and palliative chemotherapy in our cohort is consistent with randomized evidence showing limited benefit of extrapleural pneumonectomy (MARS 1) and no overall survival advantage for extended pleurectomy/decortication added to chemotherapy (MARS 2) [18, 19]. These studies underscore that optimizing systemic therapy access may yield greater population-level benefit than expanding surgical programs alone.

Updated consensus guidelines emphasize that pleurectomy/decortication should be restricted to carefully selected patients with early-stage epithelioid disease and good performance status, ideally in high-volume centers within a multimodal protocol [8]. In Latin America, such conditions are rarely met due to delayed presentation, absence of multidisciplinary mesothelioma units, and limited thoracic oncology infrastructure [12]. Consequently, surgery is performed in a small minority of patients, often reflecting access disparities rather than biological eligibility.

Immunotherapy has transformed the therapeutic landscape of MPM. The CheckMate 743 trial established nivolumab plus ipilimumab as a standard first-line option for unresectable disease, with durable benefit across histologic subtypes [3, 6]. The phase 3 IND.227/KEYNOTE-483 trial showed improved overall survival with pembrolizumab added to platinum–pemetrexed compared with chemotherapy alone, supporting chemoimmunotherapy as an additional first-line option for unresectable disease [20]. Durvalumab plus tremelimumab has also shown activity in phase 2 data, with long-term follow-up suggesting durable benefit in a subset of patients [21]. In Latin America, multicenter real-world data remain scarce, but a regional cohort treated with first-line nivolumab plus ipilimumab reported outcomes broadly consistent with trial-eligible populations, underscoring the role of access and patient selection in routine practice [22]. Trials integrating checkpoint inhibitors with chemotherapy—such as DREAM3R and BEAT-meso—suggest incremental gains in survival and continue to refine first-line strategies for advanced disease [23, 24]. Access to these agents in Latin America remains uneven, often limited to clinical trials or out-of-pocket acquisition, which likely contributes to outcome disparities [12]. Expanding national reimbursement for immune checkpoint inhibitors and ensuring supply-chain continuity are practical policy steps to narrow this gap.

### Occupational exposure and delayed asbestos dismantling

Documented exposure in our cohort included asbestos alone (15.1%) and an additional asbestos + tobacco category (27.4%), yielding > 40% of patients with recorded asbestos exposure when combined, while tobacco alone accounted for 26.4%. This distribution likely still underestimates true asbestos contact given incomplete occupational histories, informal employment, and recall limitations, but it reflects the etiologic role of asbestos in our cohort. In Latin America, the slow and uneven phase-out of asbestos has sustained environmental and occupational risk. Although Colombia’s 2019 asbestos ban marked a turning point in public health, ongoing environmental and occupational exposure persists through legacy asbestos in buildings and industrial components. Strengthening occupational surveillance, enforcing environmental decontamination, and linking exposure registries to cancer databases could substantially improve early detection. Simultaneously, addressing health-system barriers—such as delayed insurance authorization, fragmented referral pathways, and unequal access to diagnostic imaging—remains critical to improving prognosis [5, 14].

### Future directions

Future research in Latin America should focus on building collaborative registries integrating clinical, molecular, and occupational data, such as the expansion of the MeSO-CLICaP consortium [7]. Regional cooperation could facilitate prospective trials evaluating multimodal strategies adapted to local realities, and foster access to novel therapies through compassionate-use programs.

Public health policy must parallel these efforts by guaranteeing universal coverage for pemetrexed- and immunotherapy-based regimens, and by strengthening thoracic oncology training programs. Ultimately, mesothelioma in Latin America epitomizes the convergence of environmental injustice and structural inequity in cancer care; overcoming these challenges will require both scientific innovation and health-system reform [12, 14].

### Limitations

Our retrospective design and reliance on institutional records may have introduced selection bias. Data on radiotherapy dosing and quality-of-life outcomes were limited. The sample size, though one of the largest in Latin America, remains modest compared with international registries. Nonetheless, the internal consistency and alignment with prior studies support the robustness of our results.

## Conclusions

In this retrospective Colombian cohort, OS and real-world PFS were short, and baseline ECOG performance status was strongly associated with outcomes. Treatment-stratified survival curves are provided descriptively and should not be interpreted as comparative effectiveness. These findings expand regional data on pleural mesothelioma prognosis in Latin American clinical practice.

## Supplementary Information

Below is the link to the electronic supplementary material.

Supplementary file1 (DOCX 14 KB)
