# Supplementary material for: Clinical and prognostic factors in pleural mesothelioma: insights from a Latin American cohort
Source: Clin Transl Oncol. 2026 Apr 30;28(10):4839–48. doi: 10.1007/s12094-026-04337-1 (PMC13601149; doi:10.1007/s12094-026-04337-1)
Supplement: Supplementary file 1 [file 12094_2026_4337_MOESM1_ESM.docx]

**SUPPLEMENTARY MATERIAL**

**Supplementary Table S1. Proportional hazards assumption tests for multivariable Cox models.**

| **Endpoint** | **Test** | **Covariate** | **df** | **Chi-square** | **P value** |
| --- | --- | --- | --- | --- | --- |
| OS | Covariate-specific | Age (per 1-year increase) | 1 | 8.39 | 0.004 |
| OS | Covariate-specific | Male vs Female | 1 | 2.09 | 0.148 |
| OS | Covariate-specific | ECOG 2–3 vs 0–1 | 1 | 0.02 | 0.885 |
| OS | Covariate-specific | Histologic subtype | 2 | 0.26 | 0.880 |
| OS | Covariate-specific | Clinical stage | 1 | 4.63 | 0.031 |
| OS | Covariate-specific | Platelets ≥400×10^9/L vs <400×10^9/L | 1 | 0.01 | 0.943 |
| OS | **Global** | — | 7 | 13.84 | 0.054 |
| PFS | Covariate-specific | Age (per 1-year increase) | 1 | 5.33 | 0.021 |
| PFS | Covariate-specific | Male vs Female | 1 | 1.42 | 0.234 |
| PFS | Covariate-specific | ECOG 2–3 vs 0–1 | 1 | 0.04 | 0.841 |
| PFS | Covariate-specific | Histologic subtype | 2 | 1.02 | 0.599 |
| PFS | Covariate-specific | Clinical stage | 1 | 2.90 | 0.089 |
| PFS | Covariate-specific | Platelets ≥400×10^9/L vs <400×10^9/L | 1 | 1.25 | 0.264 |
| PFS | **Global** | — | 7 | 11.80 | 0.107 |

***Proportional hazards assumptions were evaluated using Schoenfeld residuals for each covariate and globally. OS indicates overall survival; PFS, progression-free survival; ECOG, Eastern Cooperative Oncology Group.***
